# The SGLT2 inhibitor dapagliflozin promotes systemic FFA mobilization, enhances hepatic β-oxidation, and induces ketosis

**DOI:** 10.1016/j.jlr.2022.100176

**Published:** 2022-02-02

**Authors:** Kristina Wallenius, Tobias Kroon, Therese Hagstedt, Lars Löfgren, Maria Sörhede-Winzell, Jeremie Boucher, Daniel Lindén, Nicholas D. Oakes

**Affiliations:** 1 Bioscience Metabolism, Research and Early Development, Cardiovascular, Renal and Metabolism, BioPharmaceuticals R&D, AstraZeneca, Gothenburg, Sweden; 2 The Lundberg Laboratory for Diabetes Research, University of Gothenburg, Gothenburg, Sweden; 3 Wallenberg Centre for Molecular and Translational Medicine, University of Gothenburg, Gothenburg, Sweden; 4 Translational Science and Experimental Medicine, Research and Early Development, Cardiovascular, Renal and Metabolism, BioPharmaceuticals R&D, AstraZeneca, Gothenburg, Sweden; 5 Functional and Mechanistic Safety, Clinical Pharmacology & Safety Sciences, R&D, AstraZeneca, Gothenburg, Sweden

**Keywords:** β-oxidation, diabetes, drug therapy, fatty acids, metabolism, tracer kinetics, SGLT2, HMG-CoA, ketone bodies, β-HBA, plasma β-hydroxybutyrate, CPT1, Carnitine palmitoyltransferase 1, HbA1c, hemoglobin A1c, *R*_*β-ox*_, Liver FFA flux into β-oxidation, *R*_*a*_, plasma FFA appearance, *R*_*ox*_, whole-body FFA oxidation, *R*_*st*_, non-oxidative FFA disposal, SGLT2, Sodium glucose cotransporter 2, TG, triglyceride

## Abstract

Sodium-glucose cotransporter 2 (SGLT2) inhibitors have been shown to increase ketone bodies in patients with type 2 diabetes; however, the underlying mechanisms have not been fully elucidated. Here we examined the effect of the SGLT2 inhibitor dapagliflozin (1 mg/kg/day, formulated in a water, PEG400, ethanol, propylene glycol solution, 4 weeks) on lipid metabolism in obese Zucker rats. Fasting FFA metabolism was assessed in the anesthetized state using a [9,10-^3^H(N)]-palmitic acid tracer by estimating rates of plasma FFA appearance (*R*_*a*_), whole-body FFA oxidation (*R*_*ox*_), and nonoxidative disposal (*R*_*st*_). In the liver, clearance (*K*_*β-ox*_) and flux (*R*_*β-ox*_) of FFA into β-oxidation were estimated using [9,10-^3^H]-(R)-bromopalmitate/[U-^14^C]palmitate tracers. As expected, dapagliflozin induced glycosuria and a robust antidiabetic effect; treatment reduced fasting plasma glucose and insulin, lowered glycated hemoglobin, and increased pancreatic insulin content compared with vehicle controls. Dapagliflozin also increased plasma FFA, *R*_*a*_, R_ox_, and *R*_*st*_ with enhanced channeling toward oxidation versus storage. In the liver, there was also enhanced channeling of FFA to β-oxidation, with increased *K*_*β-ox*_, *R*_*β-ox*_ and tissue acetyl-CoA, compared with controls. Finally, dapagliflozin increased hepatic HMG-CoA and plasma β-hydroxybutyrate, consistent with a specific enhancement of ketogenesis. Since ketogenesis has not been directly measured, we cannot exclude an additional contribution of impaired ketone body clearance to the ketosis. In conclusion, this study provides evidence that the dapagliflozin-induced increase in plasma ketone bodies is driven by the combined action of FFA mobilization from adipose tissue and diversion of hepatic FFA toward β-oxidation.

SGLT2 inhibitors, including dapagliflozin, are established treatments for patients with type 2 diabetes leading to improved glucose control as well as decreased risk of cardiovascular events and development of kidney disease (1, 2, 3, 4, 5, 6, 7). The improved cardiovascular and renal outcome data for this class of drugs have resulted in a change in standard of care recommendations, placing SGLT2 inhibitors after lifestyle interventions and metformin treatment for patients with combined diabetes and heart failure or chronic kidney disease (8).

A consistent effect of SGLT2 inhibitors in patients is an increase in plasma ketone body levels (9, 10, 11), which is presumed to be caused by increased ketogenesis. Enhanced ketogenesis may play a role in the organ protective action of SGLT2 inhibitors (12, 13, 14); therefore, understanding the nature of this phenomenon is important. The increased ketone body levels might result from a systemic increase in FFA mobilization driven by the established treatment-induced reductions in plasma glucose and insulin. Although involvement of enhanced FFA mobilization seems likely, data directly assessing this mechanism is currently lacking in the literature.

In patients with type 2 diabetes, empagliflozin (25 mg/day for 4 weeks) increased fasting ketone bodies and plasma FFA levels (15). However, the rate of appearance of glycerol, a measure of whole-body lipolysis, was not markedly altered. As described by Wolfe *et al.* (16), general changes in FFA mobilization can result not only from changes in lipolysis, but also from alterations in intra-adipocyte re-esterification of fatty acids. This could occur, for example, if reduced circulating glucose and insulin levels lower the intra-adipocyte formation of glycerol-3-phosphate leading to reduced capture of FFA released by the action of ongoing lipolysis.

An alternative explanation for the enhanced ketogenesis could be a liver-specific effect of SGLT2 inhibitors. Thus, the major site of ketone body production in the body is the liver (17), and the rate controlling enzyme for ketogenesis, at least in rodents, appears to be carnitine palmitoyl transferase 1 (CPT1) (18). The most important regulator of the activity of CPT1 is cytosolic malonyl-CoA, which is a potent CPT1 inhibitor (19). Glucose excess in the hepatocyte would tend to increase the formation and level of cytosolic malonyl-CoA, resulting in inhibition of ketogenesis. By contrast, unloading glucose from the hepatocyte would reduce malonyl-CoA removing the brake on ketogenesis.

The aim of this study was to elucidate the mechanism behind the increased ketone body levels seen following SGLT2 inhibition by measuring whole-body and tissue-specific FFA metabolism and liver co-enzyme A intermediates. Obese Zucker rats were treated with the SGLT2 inhibitor, dapagliflozin (1 mg/kg, 4 weeks), or vehicle before performing dedicated tracer studies using either [9,10-^3^H(N)]-Palmitic Acid or [9,10-^3^H]-(R)-bromopalmitate/[U-^14^C]palmitate. This study provides evidence that the dapagliflozin-induced increase in plasma ketone bodies is driven by the combined action of FFA mobilization from adipose tissue and diversion of hepatic FFA toward β-oxidation.

## Materials and methods

### Animals and treatment

Experimental procedures were approved by the local Ethics Committee for Animal Experimentation (Gothenburg region, Sweden). Male, 13-week-old obese Zucker rats (fa/fa) were purchased from Charles River Laboratories (Wilmington) and housed in an Association for Assessment and Accreditation of Laboratory Animal Care (AAALAC) accredited facility (AAALAC Unit number: 001560) with environmental control: 20–22°C, relative humidity 40–60%, with a 12-h light-dark cycle (lights off at 6 PM). The animals were housed in groups of 3–5 with free access to water and standard rodent chow (R70, Laktamin AB, Stockholm, Sweden). At 14 weeks of age, they were weighed, and a nonfasted tail vein blood sample was obtained for glucose and glycosylated hemoglobin [hemoglobin A1c (HbA1c)] analyses. Randomization into study groups was performed based on body weight and HbA1c levels. Animals were orally gavaged (2.5 ml/kg/day) at 3 PM for 4 weeks with either vehicle alone (2% ethanol, 30% PEG 400, 0.369 mg/ml propylene glycol in MQ-H2O) or dapagliflozin (1 mg/kg/day), dissolved in the vehicle. Body weight was monitored twice weekly. In all three studies the rats were given a final dose of either vehicle or dapagliflozin at 7 AM in the morning of the final experiments. Based on this protocol, three different studies were performed to examine plasma and liver biomarker responses, whole-body FFA oxidation and storage, and tissue-specific FFA utilization and storage.

#### Plasma and liver biomarker responses

Before treatment and following 4 weeks treatment, in the 16-h fasted state, tail vein blood samples were collected at 2 PM for determination of plasma biomarkers. Immediately after collection of the final tail vein sample, tissues were collected under isoflurane anesthesia. Liver samples were rapidly collected with liquid N_2_-cooled tongs for determination of glycogen and CoA intermediates. A piece of liver tissue was then collected for triglyceride (TG) analysis, and a piece of pancreas was collected for measurement of insulin and protein content. Tissues were immediately placed in N_2_-cooled tubes and placed in liquid N_2_ before storage at –80°C while awaiting analyses. Spot urine was collected at the pretreatment and 4 week time points for determination of urine glucose levels.

#### Surgical preparation

Following 4 weeks of treatment, animals were fasted overnight (from 10 PM) and the following morning (at 9 AM) they were anesthetized with Na-thiobutabarbitol dissolved in sterile water (180 mg/kg, Inactin®; RBI, Natick, MA). A tracheotomy was performed and a tube inserted to aid spontaneous breathing. One catheter was placed in the carotid artery for blood sampling and for registration of blood pressure, and two catheters were placed in the jugular vein (for tracer infusion and for topping up anesthesia as needed). Arterial catheter patency was maintained by continuous infusion (<10 μl/min) of a sterile saline solution containing sodium citrate (20.6 mM). A catheter was also placed in the bladder. Following surgical preparations, animals were allowed a 2 h stabilization period before experimental protocols commenced around 12 PM. Throughout the experiment, body temperature was monitored and maintained at 37.5°C.

#### Whole-body FFA oxidation

Whole-body FFA oxidation was determined as previously described (20). Briefly, [9,10-^3^H(N)]-palmitic acid (#NET043005MC, PerkinElmer AB, Hägersten, Sweden) was infused at a constant rate over 120 min. Repeated blood samples were collected during and after stopping the infusion for determination of unlabeled and labeled plasma FFA levels as well as ^3^H-labeled water following a lipid/water extraction procedure. Calculations of the rate of whole-body FFA oxidation (*R*_*ox*_), FFA storage (*R*_*st*_), plasma FFA clearance into oxidation (*K*_*ox*_) and storage (*K*_*st*_), and FFA appearance rate (*R*_*a*_) and clearance (*K*_*f*_) are shown in the Online Supplementary Materials. Plasma samples for analyses of glucose, insulin, and β-hydroxybutyrate (β-HBA) were collected at −5, 60, and 134 min.

#### Tissue-specific FFA oxidation

Rates of FFA utilization, storage, and oxidation were assessed based on a previously described method (21), using the partially metabolized FFA tracer, ^3^H-bromopalmitate (^3^H-R-BrP) and ^14^C-palmitate (#NEC534050UC, PerkinElmer AB). ^3^H-R-BrP was prepared by custom synthesis at Pharmaron-UK, and the purity was confirmed essentially according to methods described in (22). Briefly, both tracers were mixed and were administered as an intravenous infusion over 4 min followed by a 12 min washout before tissue collection. Repeated blood samples were collected over the 16 min study for plasma lipid extraction, determination of tracer levels, and determination of plasma FFA levels. Tissues were collected following the final blood sample for determination of the total FFA uptake (^3^H-R-BrP) and stored FFA (^14^C-palmitate). Calculations of the tissue-specific clearance and flux parameters (FFA clearance, *K*_*f*_*∗*, utilization rate, *R*_*f*_*∗*, clearance into storage, *K*_*fs*_ and rate of storage, *R*_*fs*_) are shown in the Online Supplementary Materials. From these values we also estimated the rate of (*R*_*β-ox*_) and clearance into (*K*_*β-ox*_) hepatic β-oxidation as described in the Online Supplementary Materials. The only deviation from the previously published method (21) was that the tissues in the present study were dissolved in Solvable™ (#6NE9100, PerkinElmer AB), prepared in a scintillation cocktail and then counted. Plasma samples for analyses of biomarkers were collected at −20, −5, and approximately 18 min in relation to starting the tracer infusion.

#### Blood, plasma, urine, and tissue biochemistry

Plasma glucose and blood HbA1c levels were determined from blood samples by Accu-Chek® Mobile (Roche Diagnostics, Mannheim, Germany) or the Multi-test HbA1c system (PTS Diagnostics, Hannover, Germany). Plasma FFA (#434-91795, #436-91995, Wako Chemicals GmbH, Neuss, Germany), β-HBA (#RB1007, Randox Laboratories LTD, Crumlin, UK), TG (Tri/GB, #11877771, Roche Diagnostics GmbH, Mannheim, Germany), total cholesterol (#A11A01634, HORIBA ABX, Montpellier, France), and glucose (#A11A01667, HORIBA ABX) levels were measured by enzymatic colorimetric assays on the ABX Pentra 400 (HORIBA ABX). Plasma glucagon levels were measured with an ELISA (Mercodia Glucagon ELISA, Mercodia AB, Uppsala, Sweden) and plasma insulin levels were determined using a Multi-Spot® Assay System (Meso Scale Discovery®, MD).

Tissue biomarker analysis: Liver triglycerides; A piece of liver tissue (40–60 mg) was put into a micro tube (#72.694.006, Sarstedt, Nümbrecht, Germany) with six grinding balls zirconium oxide 3 mm (#147053680090, Retsch, Haan, Germany) and homogenized in 500 μl 2-propanol in a PreCellys 24 (Bertin Technologies), for 40 s at 5,000 rpm. Another 500 μl of 2-propanol was added and samples were allowed to extract for 1 h at 4°C. Samples were then centrifuged for 5 min at 4°C at 1,300 *g*. Supernatants were analyzed using the reagent Triglycerides CP (#A11A01640, Horiba Medical). Calibrator (MultiCal, #A11A01652, Horiba Medical) and control (P-Control, #A11A01654, Horiba Medical).

Liver glycogen was measured in 70–90 mg pieces of tissue homogenized as described above but in 0.1 M acetate buffer and allowed to extract overnight at 4°C. Samples were then centrifuged and the supernatant analyzed using a 4:1 ratio of 0.5 mg/ml amyloglucosidase (#10102857 001, Roche Diagnostics) in 0.1 M acetate buffer and Glucose HK CP ABX Pentra (# A11A01667, Horiba ABX).

Pancreatic insulin content was assessed following homogenization of 40–60 mg tissue pieces in acidified ethanol (1 ml 1.5% pure HCl in 70% EtOH) and extracted overnight at −20°C. Samples were then centrifuged, and the supernatant was used for determination of insulin and total protein concentrations using a Multi-Spot® Assay System (#K152BZC, Meso Scale Discovery®) and Pierce Coomassie Plus Assay Kit (Thermo Fisher Scientific Inc, MA).

Liver malonyl CoA, acetyl CoA, succinyl CoA, and HMG CoA levels were analyzed in N_2_-freeze clamped tissue. Tissue pieces (25–50 mg) were placed in preweighed, N_2_-cooled tubes containing beads for homogenization. The frozen tube was weighed and stored at −80°C until analysis. Malonyl-, acetyl-, succinyl-, and HMG-CoA levels were analyzed by a cold, semiautomated homogenization and extraction method followed by separation by ion-pair HPLC and detection by negative electrospray tandem mass spectrometry (method described in detail in the Online Supplementary Materials).

### Statistics

Student’s *t* test, one-way or two-way ANOVA with Sidak’s multiple comparisons tests were performed using GraphPad Prism 9.0.0 (GraphPad Software Inc., La Jolla, CA). Results are reported as mean ± SD, and *P* < 0.05 was considered statistically significant.

## Results

### Dapagliflozin improves glucose control in obese Zucker rats

**Fig. 1:**
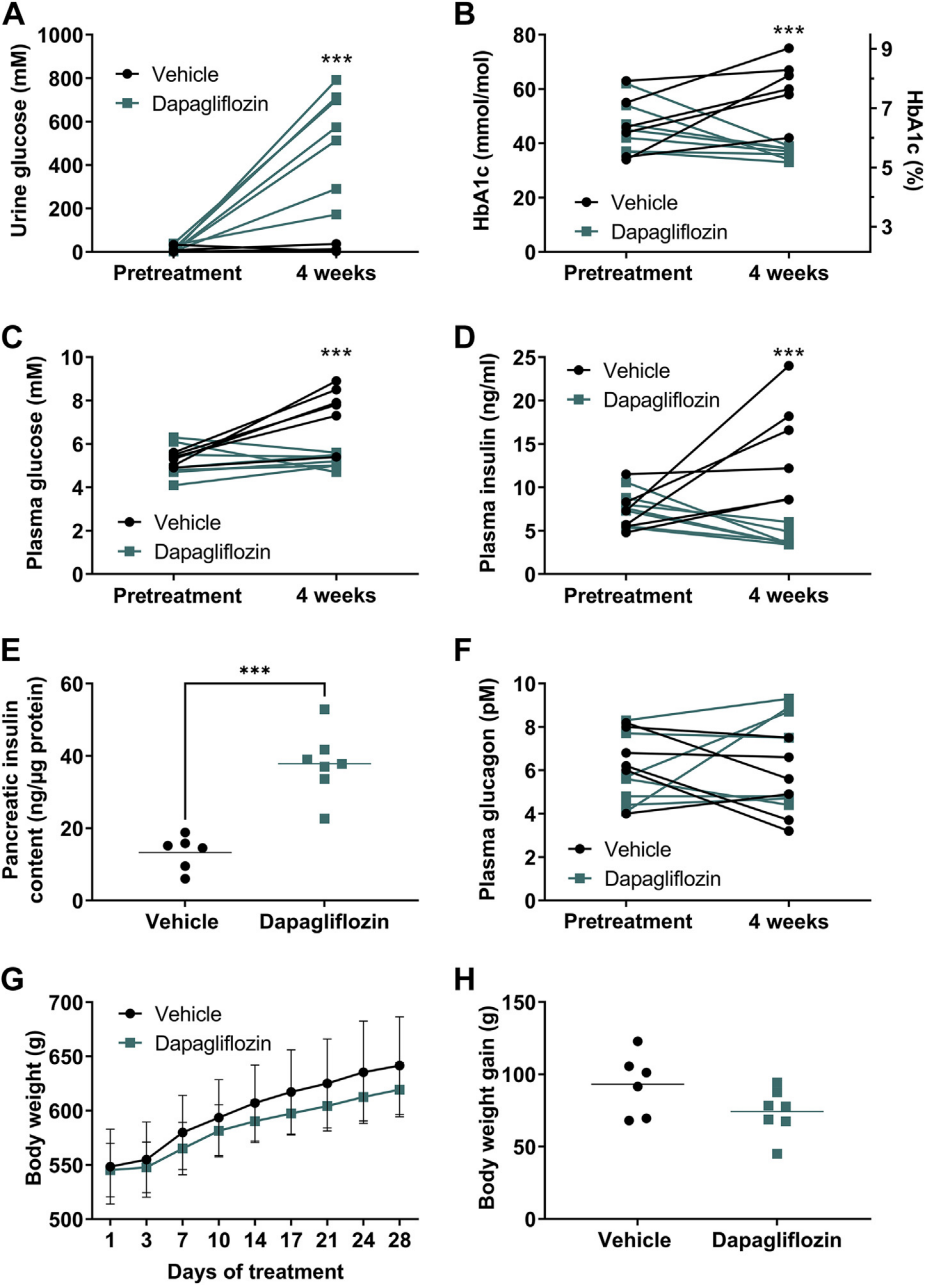
**Dapagliflozin improves glucose control in obese Zucker rats.** Male obese Zucker rats (fa/fa) were treated with either vehicle (● circles) or dapagliflozin (1 mg/kg/day, ■ squares) for 4 weeks. Pretreatment and 4 weeks treatment: (A) urine glucose levels, (B) glycosylated hemoglobin (HbA1c), 16 h fasting plasma (C) glucose and (D) insulin levels. E: Pancreatic insulin content following 4 weeks treatment. Pretreatment and 4 weeks treatment-induced 16 h fasting plasma (F) glucagon. G: Continuous body weight and (H) body weight gain during the 28 day study. Results are presented as individual data points (A–F and H) and mean ± SD (g) with n = 5–7/group: ∗∗∗*P* < 0.001; two-way ANOVA with Sidak's multiple comparisons test (A–D and F) and unpaired two-tailed *t* test (E, H).

In the obese Zucker rats, dapagliflozin treatment (1 mg/kg/day, oral gavage) for 4 weeks increased glucosuria compared with the vehicle group (Fig. 1A), consistent with expected inhibition of SGLT2. Dapagliflozin treatment also prevented the deterioration of glucose control as seen in the vehicle-treated rats (Fig. 1B, C). Thus, at the end of the treatment period, HbA1c, glucose, and insulin levels were lowered compared with vehicle control (Fig. 1B–D). The dapagliflozin treatment group also exhibited elevated pancreatic insulin content (Fig. 1E) compared with control, probably reflecting the reduced drive on insulin secretion due to reduced glycemia. These first data show, in the obese Zucker rat, that dapagliflozin treatment led to the expected metabolic effects of SGLT2 inhibition, indicating an appropriate treatment protocol and animal model to further explore the detailed metabolic consequences of dapagliflozin treatment. Treatment did not affect fasting plasma glucagon levels compared with vehicle control (Fig. 1F). There was a tendency for reduced body weight gain in dapagliflozin- versus vehicle-treated rats; however, statistical significance was not reached (Fig. 1G, H). Also, liver glycogen and TG contents were not significantly different between groups (supplemental Fig. S1A, B). Plasma FFA and β-HBA levels were increased following dapagliflozin treatment (supplemental Fig. S1C, D); however, plasma TG or total cholesterol levels were not different from vehicle (supplemental Fig. S1E, F).

### Dapagliflozin increases FFA supply, whole-body and liver FFA oxidation

**Fig. 2:**
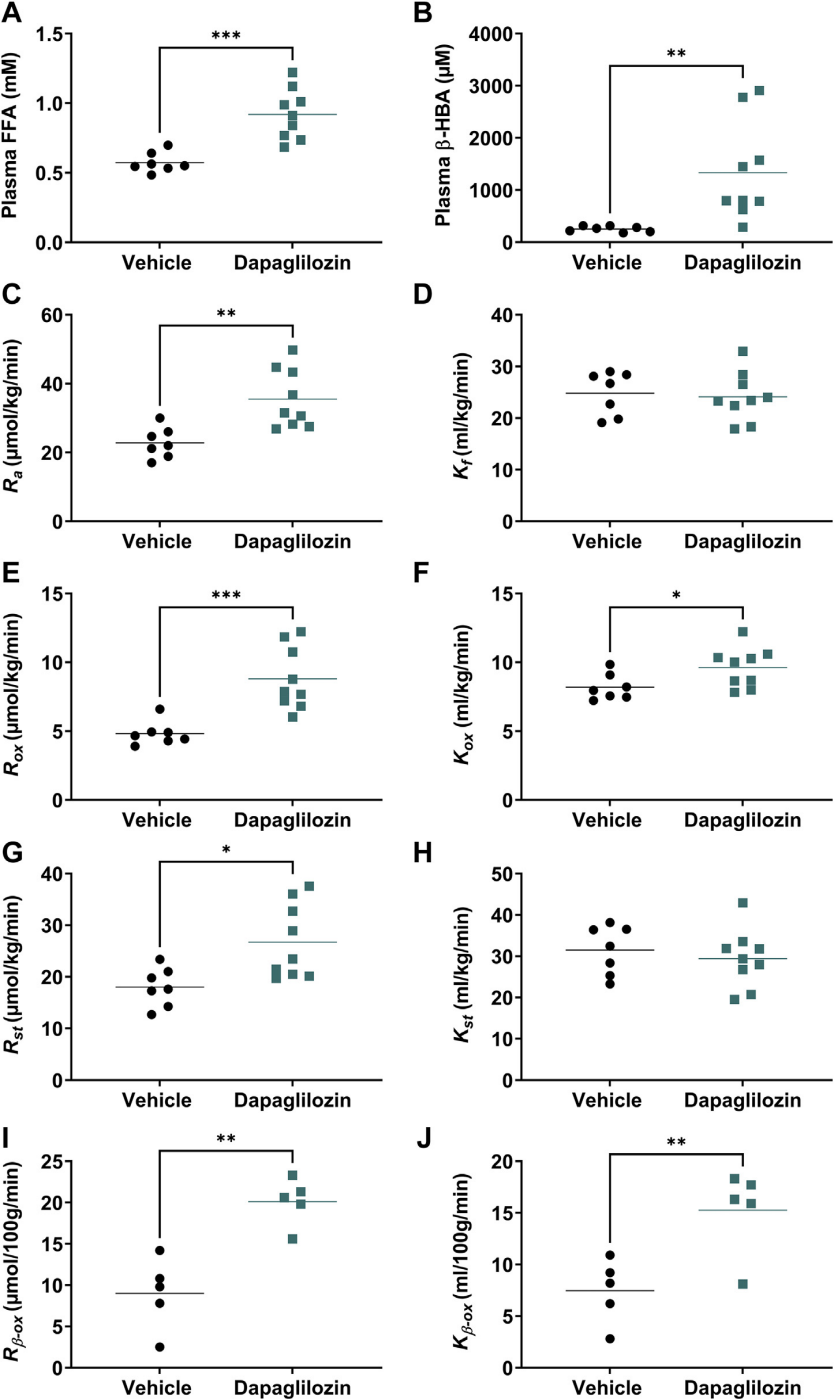
**Dapagliflozin increases FFA supply, whole-body and liver FFA oxidation.** Assessment of (A–H) whole-body FFA oxidation, using a ^3^H-palmitate tracer, or (I, J) liver-specific FFA oxidation, using ^3^H-Br and ^14^C-palmitate tracers, in male obese Zucker rats (fa/fa), pretreated with either vehicle (● circles) or dapagliflozin (1 mg/kg/day, ■ squares) for 4 weeks with measurements made in the 16 h fasted, anesthetized state. Plasma (A) FFA, (B) β-hydroxybutyrate (β-HBA) and calculated (C) FFA appearance rate (*R*_*a*_), (D) FFA clearance (*K*_*f*_), (E) whole-body FFA oxidation rate (*R*_*ox*_), (F) whole-body FFA clearance into oxidative metabolism (*K*_*ox*_), (G) whole-body FFA storage rate (*R*_*st*_), (H) whole-body FFA clearance into storage (*K*_*st*_), (I) liver FFA flux into oxidation (*R*_*β-ox*_), and (J) liver FFA clearance into oxidation (*K*_*β-ox*_). All data presented as individual data points with group means, n = 5–9/group: ∗*P* < 0.05; ∗∗*P* < 0.01; ∗∗∗*P* < 0.001, by unpaired two-tailed Students’ *t* test.

In the following tracer studies, measurements were performed in fasted, anesthetized animals following the same pretreatment protocol as applied in the first study. As a result of 4 weeks’ treatment with dapagliflozin, fasting plasma FFA and β-HBA levels were increased compared with vehicle-treated animals (Fig. 2A, B). Other plasma biomarkers measured during the tracer studies are presented in supplemental Table S1. The elevation of FFA levels could be completely accounted for by an increased FFA mobilization: dapagliflozin increased FFA appearance rate (*R*_*a*_, Fig. 2C) while not changing the plasma FFA clearance (*K*_*f*_) compared with controls (Fig. 2D). FFA disposal was resolved into oxidative and nonoxidative pathways. Whole-body FFA oxidation rate (*R*_*ox*_) was significantly increased after dapagliflozin treatment compared with vehicle control (Fig. 2E), due to a specific enhancement in oxidative clearance (*K*_*ox*_, Fig. 2F), as well as increased substrate availability (Fig. 2A). Dapagliflozin also increased nonoxidative disposal (*R*_*st*_, Fig. 2G); however, in contrast to the oxidative pathway, this was completely driven by an increase in substrate availability, with no effect on nonoxidative clearance (*K*_*st*_, Fig. 2H).

To identify the tissue locus responsible for the observed whole-body effect on FFA oxidation, we assessed the FFA utilization index (*R*_*f*_*∗*), which includes oxidation and storage, as well as a measure reflecting only FFA storage (*R*_*fs*_), in an extensive range of tissues (supplemental Table S2). Enhanced channeling of FFA into β-oxidation (*R*_*β-ox*_) in a specific tissue would result in a relative decrease in the ratio *R*_*fs*_/*R*_*f*_∗ in that tissue. This only occurred in one tissue, the liver (supplemental Table S2). Furthermore, estimation of hepatic β-oxidation rate (*R*_*β-ox*_), from the *R*_*f*_*∗* and *R*_*fs*_ values (as described in supplemental materials), revealed a marked increase in dapagliflozin compared with vehicle controls (Fig. 2I). The increase in *R*_*β-ox*_ involved a large enhancement in hepatic oxidative clearance (*K*_*β-ox*_) of >2-fold in the dapagliflozin- compared with vehicle-treated group (Fig. 2J). In fact, taking into account the liver weight, we estimate that the liver could quantitatively account for the observed increase in whole-body FFA oxidation. Thus, using average liver weights of 4.56 and 4.16% of body weight in vehicle and dapagliflozin groups, respectively, (based on measurements in the first cohort of animals) and the tissue weight-corrected *R*_*β-ox*_ values (Fig. 2I), the liver contribution to the whole-body FFA oxidation is estimated to be 4.1 and 8.4 μmol/kg/min in vehicle and dapagliflozin groups, respectively, a difference of 4.3 μmol/kg/min. This matches very closely the difference in *R*_*ox*_ of 4.0 μmol/kg/min (Fig. 2E). Together, these data show that dapagliflozin enhanced whole-body FFA oxidation through a specific effect in the liver.

Interestingly, the ratio R_*fs*_/R_*f*_∗ was increased in the renal cortex (supplemental Table S2) consistent with the location of the primary target, SGLT2. This is possibly consistent with a reduction in FFA oxidation, resulting from a reduced requirement to support salt and glucose reabsorption.

### Dapagliflozin-induced changes in liver substrates during the fasted state support elevated ketogenesis

**Fig. 3:**
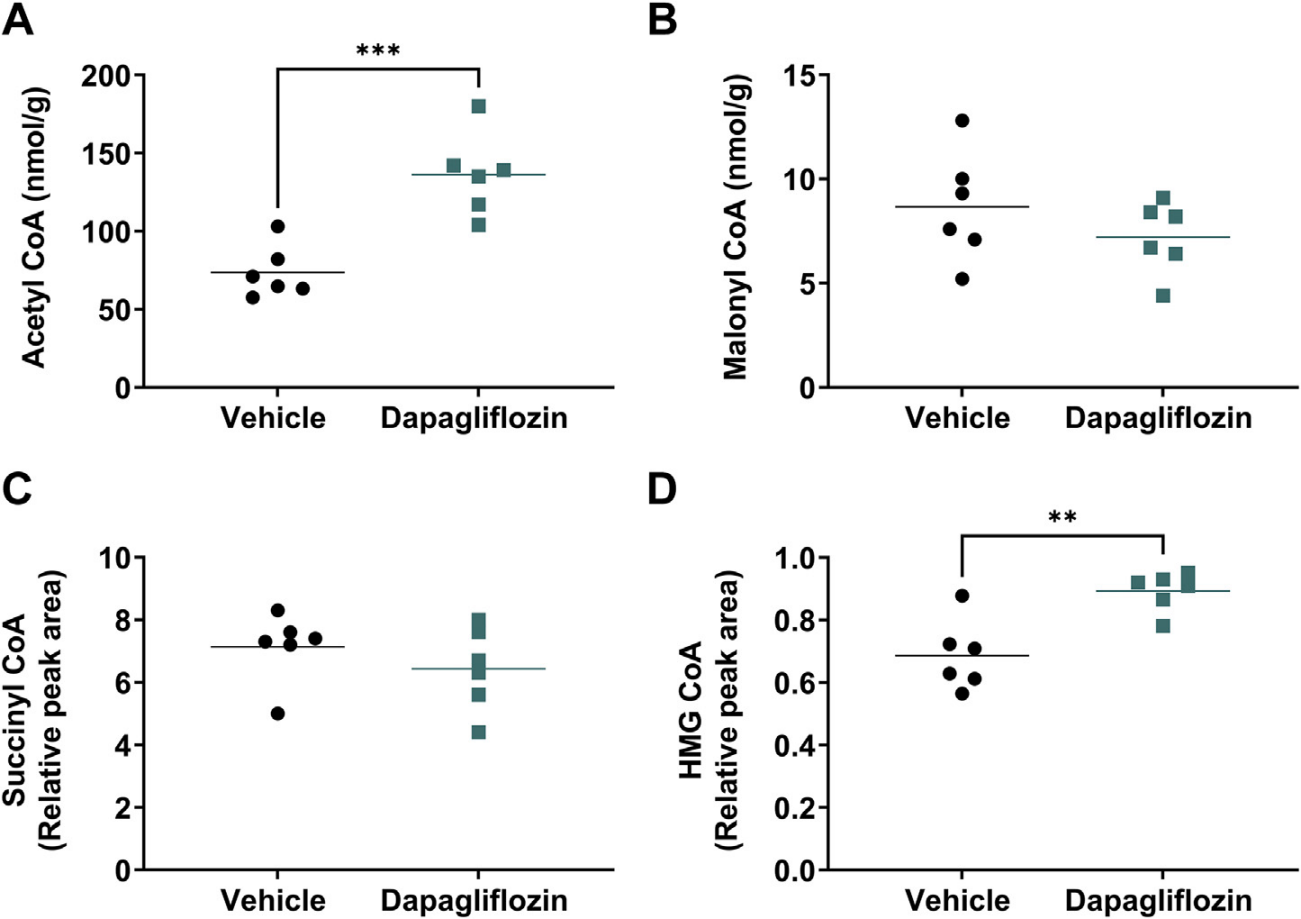
**Dapagliflozin-induced changes in liver substrates favor ketone body formation.** Liver tissue levels in the 16 h fasted state of (A) Acetyl CoA, (B) Malonyl CoA, (C) Succinyl CoA, and (D) HMG CoA, following 4 weeks treatment in male obese Zucker rats (fa/fa), with either vehicle (● circles) or dapagliflozin (1 mg/kg/day, ■ squares). All data presented as individual values with group means, n = 6–7/group: ∗∗*P* < 0.01; ∗∗∗*P* < 0.001, by unpaired two-tailed Students *t* test.

Liver acetyl-CoA, the major product of β-oxidation and precursor for ketogenesis and the tricarboxylic acid cycle, was elevated in the dapagliflozin-treated rats compared with vehicle (Fig. 3A). Although mean levels of malonyl-CoA, a master regulator of FFA uptake into the mitochondria for oxidation and a building block for de novo lipogenesis, tended to be lower in the dapagliflozin-treated group, statistical significance was not achieved (Fig. 3B). The tricarboxylic acid cycle intermediate succinyl-CoA was not different between treatment groups (Fig. 3C). In contrast, HMG-CoA, an intermediate in ketone body formation, was significantly increased in the livers of the dapagliflozin-treated rats compared with vehicle (Fig. 3D) and consistent with elevated ketogenesis and the increased plasma β-HBA levels, observed in the dapagliflozin-treated rats compared with vehicle (Fig. 2B, supplemental Fig. S1D and supplemental Table S1).

## Discussion

**Fig. 4:**
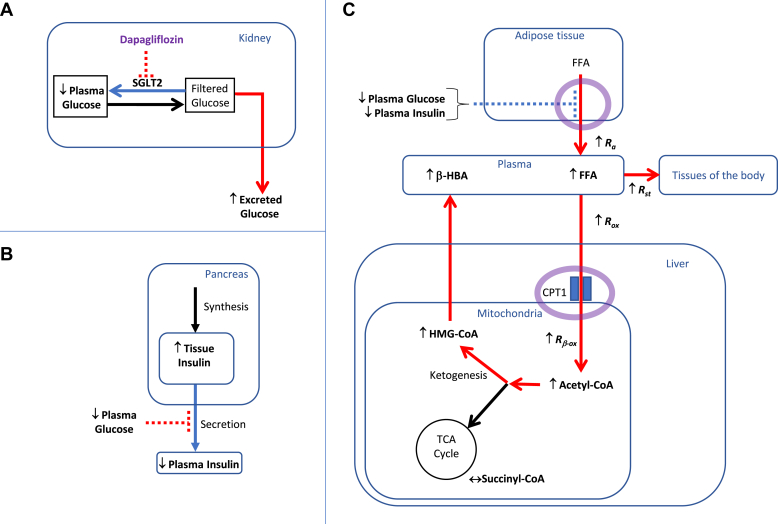
**Summary of metabolic effects of dapagliflozin in the fasting state.** A: Prevention of hyperglycemia is achieved by the glycosuria resulting from the primary pharmacodynamic action of dapagliflozin, inhibition of the SGLT2 in the renal tubules. B: The antihyperglycemic action of dapagliflozin reduces the insulin secretory burden resulting in reduced levels of plasma insulin and increased pancreatic insulin content. C: The antihyperglycemic and antihyperinsulinemic effects of dapagliflozin likely drove the observed whole-body FFA mobilization from adipose tissue (↑*R*_*a*_) and elevated systemic FFA availability (↑FFA). Dapagliflozin enhanced whole-body FFA oxidation (↑*R*_*ox*_), an effect that could be completely accounted for by a liver-specific induction of β-oxidation (↑*R*_*β-ox*_). Elevated β-oxidation increased hepatic acetyl-CoA levels and drove ketogenesis, evidenced through elevations in liver tissue HMG-CoA and plasma β-HBA. Solid lines represent fluxes, with dapagliflozin-induced changes in flux represented by color: blue, decreased; black, unchanged; and red, increased flux. Dotted lines represent equivalent inhibitory control signals, with dapagliflozin-induced changes in the strength of inhibition represented by color: blue, decreased and red increased inhibition.

Consistent with effects seen in multiple clinical trials in patients with type 2 diabetes (23), in the current study, SGLT2 inhibitor treatment improved glucose control in the obese Zucker rat. The current results also confirmed that dapagliflozin elevates fasting levels of the ketone body, β-HBA, as seen in the clinic (9). Putting our data together suggests that dapagliflozin promotes ketogenesis via the mechanisms summarized in Fig. 4. In line with inhibition of the primary target, SGLT2, dapagliflozin promoted glucosuria and decreased plasma glucose (Fig. 4A). The reduced glycemia probably led to lower insulin secretion, resulting in the observed reduction in plasma insulin levels and the increased pancreatic insulin content (Fig. 4B). These results are consistent with established effects of SGLT2 inhibitors (24). In the present study, comprehensive tracer methods were then applied to reveal novel insights into the mechanism of SGLT2 inhibitor action upon in vivo whole-body and tissue-specific FFA fluxes (Fig. 4C). The antihyperglycemic and antihyperinsulinemic effects of dapagliflozin likely drove the observed whole-body FFA mobilization from adipose tissue (*R*_*a*_) and elevated systemic FFA availability. Enhanced whole-body FFA oxidation (*R*_*ox*_) could be completely accounted for by a liver-specific induction of β-oxidation (*R*_*β-ox*_). Elevated β-oxidation increased hepatic acetyl-CoA levels and drove ketogenesis, evidenced through elevations in liver tissue HMG-CoA and plasma β-HBA. Recent publications following dapagliflozin treatment of patients with type 2 diabetes support the translational value of this study as increased whole-body fatty acid oxidation, and specifically increased hepatic FFA uptake, was observed in these clinical trials (25, 26).

The enhanced FFA mobilization could theoretically be due to increased lipolysis resulting from a reduction in plasma insulin or a decrease in intra-adipocyte re-esterification, due to falling glucose and insulin levels. Of these alternatives the available evidence suggests that reduced re-esterification is the dominant driver since in the clinic another SGLT2 inhibitor has been shown to minimally influence lipolysis (15).The lack of change in plasma glucagon levels with dapagliflozin treatment in the current study (Fig. 1F) adds to evidence that glucagon signaling is not responsible for the increased ketogenesis. While several studies have shown that SGLT2 inhibitors increase plasma glucagon levels in patients with type 2 diabetes (24, 27, 28), this does not appear to be the case in people with prediabetes (29). Furthermore, a recent publication, based on studies in mice, provides strong evidence that SGLT2 inhibitor-mediated enhancement of ketogenesis is independent of glucagon signaling (30).

Our data reveal that dapagliflozin robustly enhances β-oxidation in the liver by a local, liver-specific effect. The rate-limiting step for this process is thought to be long-chain fatty acid flux into the mitochondria via CPT1 (31, 32). The main allosteric regulator of this enzyme is malonyl-CoA (19) and increases in plasma glucose and insulin levels elevate hepatic malonyl-CoA levels (33, 34). We were therefore expecting a clear treatment-induced reduction in hepatic malonyl-CoA, and although mean levels tended to be lower in the dapagliflozin-treated versus vehicle-treated animals (Fig. 3B), this did not reach statistical significance. A key factor responsible for the enhanced hepatic CPT1 activity may have been the insulin lowering effect of dapagliflozin. Thus insulin, per se, time dependently increases the sensitivity to inhibition of CPT1 by malonyl-CoA (35). Conversely, progressive insulin lowering occurring during prolonged fasting may be responsible for delayed reduction in the sensitivity of CPT1 to the inhibitory influence of malonyl-CoA (36). Thus, the chronic dapagliflozin-induced reduction in plasma insulin levels, by decreasing the sensitivity of CPT1 to the inhibitory influence of malonyl-CoA and thereby enabling higher FFA flux into the mitochondria, may have been the major driver for the observed enhancement in hepatic β-oxidation.

Dapagliflozin promoted the mobilization of FFA, which drove an increase in nonoxidative disposal of FFA (Fig. 2G). Preformed fatty acids taken up from the circulation are a quantitively important source of liver lipid (37). Despite the dapagliflozin-induced increase in systemic FFA availability, hepatic TG content was not increased by treatment (supplemental Fig. S1B). This implies that the increased delivery of plasma FFA to this storage pool was being offset either through increased hepatic TG pool turnover or reduced local de novo lipogenesis, the other major source of the fatty acid moiety of TG. Indeed, both of these offsetting factors are likely to have been engaged. Thus, decreased de novo lipogenesis has not been verified but would certainly be expected given the reductions of glycemia and insulin levels due to their strong transcriptional regulation of this pathway (38). In addition, the observed enhancement in β-oxidation would be expected to increase turnover of the local TG pool. We were actually surprised that dapagliflozin did not significantly lower hepatic lipid, as has been observed in patients with type 2 diabetes following 8 weeks of treatment (39). The hepatic TG pool of the obese Zucker rat is large, and whether this was due to an insufficient treatment duration of only 4 weeks would require answering with new experiments.

Dapagliflozin successfully prevented the development of diabetes in the initially prediabetic obese Zucker rats used in this study. In addition to preventing the deterioration in glucose control, as seen in the vehicle-treated animals, dapagliflozin treatment also increased pancreatic insulin content (Fig. 1E), in line with previous observations in db/db mice (19). If translatable to the clinic, this possible β-cell sparing effect has the potential to extend treatment to patients with prediabetes, at high risk of developing type 2 diabetes. Indeed, a study of overweight women who had recently experienced gestational diabetes showed that dapagliflozin in combination with metformin had greater beneficial effects on glycemic and metabolic parameters compared with metformin alone (40). These beneficial effects included an improvement in early insulin response to a glucose challenge, consistent with an increase in pancreatic insulin content.

Although many of the results tie together and confirm results in earlier clinical studies using SGLT2i, there are a number of limitations of the current work. First, all of our studies are restricted to the fasting situation. This was motivated by our primary interest in exploring the mechanisms of ketogenesis, which are suppressed by the physiological actions of hyperinsulinemia and hyperglycemia. There are also two major species of ketone bodies in the circulation, β-HBA and acetoacetate. In this study we have only measured β-HBA, as results from a pilot study suggested that plasma acetoacetate was not stable under the conditions of plasma handling and storage applied. Furthermore, we have not directly assessed ketone body production and clearance. Therefore, we cannot exclude the possibility that a reduction in the clearance rate of plasma ketone bodies contributes to the dapagliflozin-induced elevation of plasma ketone bodies observed. The interpretation of liver CoA intermediate levels is complicated by the unknown partitioning of the metabolites between subcellular compartments: e.g., mitochondrial HMG-CoA is destined for ketogenesis while the cytosolic pool can be directed to de novo cholesterol synthesis. A lack of change in plasma cholesterol (supplemental Fig S1F), however, argues against an involvement of the cytosolic pool in the observed increase in total HMG-CoA levels. The tracer studies were also performed in the anesthetized state, which can of course perturb metabolism; however, this is unlikely to have influenced our major conclusions. Thus, the relative differences between dapagliflozin- and vehicle-treated groups in plasma FFA and β-HBA were maintained. Another limitation of the current methodology is that the turnover and metabolic fate of esterified FA have not been assessed. Thus, our measurements of FFA oxidation exclude the contribution of FAs derived from hydrolysis of intracellular TGs. Finally, small amounts of ethanol were used to formulate the dapagliflozin for oral administration as well as administration of the FFA tracer; however, both the vehicle and dapagliflozin groups received equal per kg doses of ethanol. Although ethanol can potentially perturb ketone body metabolism (41, 42), the dosing needed to achieve these effects is much higher than the ones used in the present study.

In conclusion, this study provides new insight into the mechanisms by which an SGLT2 inhibitor, dapagliflozin, enhances ketogenesis. Our tracer kinetic studies in the fasting state provide evidence that the ketogenic effect is driven by the combination of accelerated mobilization of FFA from adipose tissue stores and a robust liver-specific enhancement in β-oxidation. These responses qualitatively resemble the physiologic response to fasting. Thus, faced with a situation of negative carbohydrate balance, due to SGLT2 inhibition and glycosuria, the decreasing glucose and insulin levels are probably primary triggers via established physiologic mechanisms for the observed adipose and hepatic lipid metabolic responses.

## Data availability

All data are available in the main text or the supplementary materials.

## Supplemental data

This article contains supplemental data (20, 21).

## Conflict of interest

All authors are or were employed by AstraZeneca and have shares in the company.
