# Supplementary material for: The SGLT2 inhibitor dapagliflozin promotes systemic FFA mobilization, enhances hepatic β-oxidation, and induces ketosis
Source: J Lipid Res. 2022 Feb 2;63(3):100176. doi: 10.1016/j.jlr.2022.100176 (PMC8953658; doi:10.1016/j.jlr.2022.100176)
Supplement: Online supplementary material [file mmc1.docx]

**Supplementary material**

**Supplementary Methods**

UPLC-tandem-MS method for quantification of acyl-CoA in tissues samples

Malonyl-, acetyl-, succinyl- and HMG-CoA levels in tissues were analyzed by a cold, semi-automated homogenization and extraction method followed by separation by ion-pair liquid chromatography and detection by negative electrospray tandem mass spectrometry.

About 50 mg (30-70 mg) tissue was snap frozen and collected in pre-weighed cold empty 2 ml reinforced homogenization tubes (Precellys, #KT03961-1-403.2) and stored at -80 °C until analyzed. To each tissue-tube, as well as to separate tubes containing 50 µl of calibration sample, 6 µl/mg (*i.e.* 300 µl to 50 mg tissue or 50 µl to the 50 µl calibration sample) of cold (-20 °C) extraction buffer (40% methanol, 5% trifluoroacetic acid, 0.1% dithiotreitol, spiked with 10% of 15 µM ^13^C-malonyl-CoA internal standard solution), 50 µl 30% bovine serum albumin in phosphate buffered saline (Sigma, #A9576–50ML) and 0.5 ml ice-cold chloroform was added followed by the addition of *n* = 3-6 cold (4°C) yttrium-stabilized zirconium oxide beads (Retsch, #05.368.0090).

All tubes were stored at -20 °C for 30 minutes prior the simultaneous and automated batch homogenization and initial extraction with the Precellys-24 (Bertin, France) performed for 2×20 s at power 5000. Complete extraction and sample purification (protein precipitation and removal of lipids) was performed for 5 min at 25 Hz using a Mixer Mill 300 (Retsch, Germany). Following centrifugation (3000G, 10 min), 150 µl of the upper aqueous sample extract was transferred to glass vials and evaporated to dryness at 50 °C under a stream of nitrogen. Analytes were dissolved in 100 μL injection solvent (0.5% trifluoroacetic acid, 2% acetonitrile in water).

Extracts was analyzed by a UPLC-tandem-MS system (injecting 2 µl). Separation was achieved in 8 min using a binary solvent system and a gradient run (0% B from 0.0–0.2 min; 0-100% B from 0.2-3.0 min; kept at 100% B for 2.5 min; returned to 0% B from 5.6-7.6 min) where phase A contains 10 mM ammonium acetate, 5 mM acetic acid, 10 mM diisopropylamine and 2% acetonitrile in water and phase B contains 25% phase A in acetonitrile. The column was a Kinetex 1.7 µm EVO C18 50 x 2.1 mm (Phenomenex, #00B-4726-AN).

All analytes were determined by negative electrospray tandem-mass spectrometry using the Acquity UPLC and Xevo TQS tandem mass spectrometer (Waters). The electrospray source was operated at 150 °C, with the capillary at 2.0 kV, desolvation gas at 500 °C, cone gas flow at 50 L/hour and the desolvation gas flow at 1000 L/hour. The waste valve solvent delay was on from 0–2 min and 4–5 min. The mass spectrometer was operated in multiple reaction monitoring mode with a cone voltage of 50 kV and dwell time of 0.050 s for all analytes. The mass transitions were 852 > 808 (malonyl-CoA), 855 > 810 (^13^C_3_–malonyl-CoA), 808 > 408 for acetyl-CoA, 866 > 408 for succinyl-CoA and 910 > 408 for HMG-CoA. The collision energy was 25 eV (malonyl-CoA and ^13^C_3_–malonyl-CoA) or 35 eV (acetyl-CoA, succinyl-CoA and HMG-CoA).

For quantitative analysis, tissue sample results were calibrated against the ^13^C-malonyl-CoA internal standard internal standard for each of malonyl-, acetyl-, succinyl- and HMG-CoA and as well against an external 5-point calibration curve ranging from 160 nM to 100 μM for malonyl CoA and 320 nM to 200 µM for acetyl-CoA, prepared in extraction buffer diluted 10 times with 40% methanol and stored at −80 °C, and allowing for additional absolute quantitation of malonyl-CoA and acetyl-CoA. Tissue samples were processed the same way as calibration samples through the sample preparation and LC-MS process and thus absolute concentrations of malonyl- and acetyl-CoA and relative concentrations for succinyl- and HMG-CoA in HPLC extracts were given directly from comparison of the peak responses with the calibration curves for malonyl- and acetyl-CoA and for the peak response relative to the internal standard peak response for succinyl- and HMG-CoA. Concentrations in tissue samples are calculated by normalizing the actual sample amount to the nominal amount of 50 mg (the amount of calibration sample). The detection limits were < 100 pmol/g wet weight tissue.

All stock solutions of malonyl-^13^C_3_-coenzyme A lithium salt (Isotec #65,575-9, MW 862.5), malonyl coenzyme A lithium salt (Sigma, #M4263, MW 859.5, CAS 108347-84-8), acetyl coenzyme A trilithium salt (Sigma, #A2181, MW 827.4, CAS 75520-41-1), succinyl coenzyme A sodium salt (Sigma, #S1129, MW 867.6, CAS 604-98-8) and HMG-coenzyme A (Sigma, #H6132, MW 1009, CAS 1553-55-5) and IS solutions and calibration samples were prepared in a reference buffer made of 40 % methanol with 0.5 % trifluoracetic acid and 0.001% dithiotreitol, prepared by dilution 10 times of the extraction buffer with 40% methanol. Stock solutions and the calibration samples were stored in 5 ml amber borosilicate glass vials or 20 ml liquid scintillation vials (IS) at –80 °C, thawed and pipetted cold (4 °C).

**Calculations**

**Whole body FFA oxidation:**

Rates of plasma FFA clearance and appearance were assessed using a constant infusion of ^3^H-palmitate (^3^H-P) (1). At steady state (<40 min after the start of tracer infusion), the plasma clearance rate of ^3^H-P (*K_f_*) was calculated as:

$K_{f}=\frac{i_{p}}{c_{p (\infty)}}$

where *i_p_* is the tracer infusion rate (dpm min^-1^) and *c_p_* (*∞*) is the steady state arterial concentration of ^3^H-P (dpm/ml). The rate of appearance of plasma FFAs (*R_a_*) was calculated as:

$R_{a}=C_{p}\times K_{f}$

where *C_p_* is the arterial plasma FFA concentration (mM).

An estimate of the whole-body clearance of FFA into oxidation (*K_ox_*) was calculated from the plasma accumulation of ^3^H_2_O as:

$K_{ox}=V_{w}\times\frac{dc_{w}}{dt}\times\frac{1}{c_{p}(\infty)}$

where *V_w_* is the total water space estimated from the regression equation: % body water = 59.3-0.027*body weight (g), relating percent measured body water content vs body weight of obese Zucker rats. *c_w_* is the plasma concentration of ^3^H_2_O and *t* is the time from commencement of tracer infusion. The derivative above was estimated from the slope obtained from linear regression analysis of the ^3^H_2_O plasma versus time data for the period *t* = 0 to *t* = 120 min.

The whole-body rate of fatty acid oxidation *R_ox_* was calculated as:

$R_{ox}=C_{p}\times K_{ox}$

Whole body FFA clearance into storage metabolism was calculated as:

$K_{st}=K_{f}-K_{ox}$

The corresponding rate of storage is calculated as:

$R_{st}=C_{p}\times K_{st}$

### Tissue-specific fatty acid oxidation:

*Tissue-specific FFA clearance and utilization rate indices*

The clearance rate of ^3^H-R-BrP by an individual tissue (*K_f_**) is an index of the ability of the tissue to utilize FFAs and was calculated as described in detail in Oakes et al. (2) and briefly below:

$K_{f}^{*}=\frac{m_{B}}{\int_{0}^{T} c_{B}(t)dt}$

where *T* is the time of tissue collection, *m_B_* is the total tissue ^3^H content (at *t* = *T*), *c_B_* is the arterial plasma concentration of ^3^H-R-BrP.

An index of FFA utilization rate (*R_f_**) was calculated as:

$R_{f}^{*}=C_{P}\times K_{f}^{*}$

where *C_P_* is the arterial plasma FFA concentration.

An index of the clearance of ^14^C-P into storage products (*K_fs_*) was calculated as:

$K_{fs}=\frac{m_{P}}{\int_{0}^{T} c_{P}(t)dt}$

where *m_P_* is the total tissue ^14^C-content (at *t*=*T*) and *c_P_* is the arterial plasma ^14^C-P concentration.

An index of the rate of FFA incorporation into storage (*R_fs_*) was calculated as

$R_{fs}=C_{P}\times K_{fs}$

An estimate of the rate of hepatic β-oxidation (*R_β-ox_*) can be made as follows:

$R_{\beta-ox}={R_{f}^{*}}/{LC^{*}-R_{fs}}$

Where *LC^*^* is the lumped constant for the liver (0.79), defined as the ratio of *R_f_^*^* to true FFA β-oxidation rate (*R_β-ox_*).

**Supplemental results**

| **Supplemental Table S1.** Residual data corresponding to the whole-body FFA oxidation experiment (related to Fig. 2a-h) and tissue-specific FFA oxidation experiment (related to Fig. 2i-j and Supplemental Table S2), in overnight fasted, anesthetized obese Zucker rats (fa/fa) following 4 weeks treatment with either vehicle or dapagliflozin (1 mg/kg/day). | | | |
| --- | --- | --- | --- |
| ***Whole-body FFA oxidation experiment*** | **Vehicle** (*n*=7) | **Dapagliflozin** (*n*=9) | *P value* |
| *Basal* HbA1c (mmol/mol) | 56±7 | 56±10 | 0.996 # |
| *4 weeks* HbA1c (mmol/mol) | 66±19 | 46±9 | 0.004 # |
| Plasma insulin (ng/ml) | 15.99±4.73 | 10.53±4.86 | 0.041 |
| Plasma glucose (mM) | 12.01±4.32 | 8.66±2.63 | 0.067 |
| Body weight gain (g/24 days) | 72.69±25.77 | 51.96±21.8 | 0.073 |
|  |  |  |  |
| ***Tissue-specific FFA oxidation experiment*** | **Vehicle** (*n*=5) | **Dapagliflozin** (*n*=5) | *P value* |
| *Basal* HbA1c (mmol/mol) | 41±4 | 39±3 | 0.905 # |
| *4 weeks* HbA1c (mmol/mol) | 55±17 | 37±5 | 0.019 # |
| Plasma insulin (ng/ml) | 18.3±4.3 | 7.46±1.24 | 0.0006 |
| Plasma glucose (mM) | 13.14±3.39 | 9.5±1.44 | 0.058 |
| Plasma lactate (mM) | 1.52±0.24 | 0.84±0.15 | 0.0007 |
| Plasma FFA (mM) | 1.16±0.17 | 1.39±0.33 | 0.203 |
| Plasma betahydroxybutyrate (µM) | 820±365 | 2626±1622 | 0.041 |
|  |  |  |  |
| Data presented as mean ± SD. ^#^ *P* value obtained from Sidak's multiple comparisons test following a two-way analysis of variance (ANOVA). All other comparisons by unpaired, two-tailed Student's T-test. | | | |

| **Supplemental Table S2.** Tissue-specific FFA utilization (*R_f_**), storage rate (*R_fs_*) and the ratio between R*_fs_* and R*_f_** in overnight fasted, anesthetized obese Zucker rats (fa/fa) following 4 weeks treatment with either Vehicle or Dapagliflozin (1 mg/kg/day) | | | | | | | | | |
| --- | --- | --- | --- | --- | --- | --- | --- | --- | --- |
|  | ***R_f_** (µmol/100g/min)** | |  | ***R_fs_* (µmol/100g/min)** | |  | ***R_fs_/R_f_**** | |  |
| **Tissues** | **Vehicle** | **Dapagliflozin** |  | **Vehicle** | **Dapagliflozin** |  | **Vehicle** | **Dapagliflozin** |  |
| RQ | 1.96±0.42 | 2.36±0.59 |  | 5.38±1.2 | 6.19±1.52 |  | 2.75 ± 0.31 | 2.73 ± 0.68 |  |
| WQ | 0.48±0.03 | 0.59±0.13 |  | 1.25±0.38 | 1.46±0.46 |  | 2.61 ± 0.80 | 2.50 ± 0.51 |  |
| EAT | 0.75±0.27 | 1.14±0.33 |  | 1.81±0.99 | 2.69±1.08 |  | 2.28 ± 0.66 | 2.28 ± 0.26 |  |
| IAT | 0.76±0.19 | 1.33±0.73 |  | 1.91±0.73 | 3.03±0.89 |  | 2.47 ± 0.47 | 2.31 ± 0.78 |  |
| BAT | 3.04±0.52 | 3.03±0.62 |  | 5.39±1.36 | 5.78±1.48 |  | 1.77 ± 0.35 | 1.83 ± 0.29 |  |
| Diaphragm | 3.5±0.53 | 3.99±0.8 |  | 8.43±1.21 | 11.03±1.6 |  | 2.42 ± 0.29 | 2.88 ± 0.53 |  |
| Heart | 13.15±2.54 | 11.51±6.61 |  | 10.6±1.67 | 13.06±0.75 |  | 0.83 ± 0.22 | 1.70 ± 0.1.48 |  |
| Kidney C | 4.98±1.02 | 4.72±0.91 |  | 12.27±1.09 | 14.97±2.14 |  | 2.54 ± 0.51 | 3.18 ± 0.25* |  |
| Kidney M | 3.87±0.53 | 4.33±0.51 |  | 10.39±0.89 | 13.65±1.78 |  | 2.74 ± 0.54 | 3.15 ± 0.34 |  |
| Liver | 32.95±7.32 | 44.92±7.58 |  | 32.69±7.27 | 36.75±10.74 |  | 1.00 ± 0.11 | 0.78 ± 0.15* |  |
| Red quadriceps (RQ), White quadriceps (WQ), Epididymal adipose tissue (EAT), Inguinal adipose tissue (IAT), Brown adipose tissue (BAT), Kidney cortex (Kidney C), Kidney medulla (Kidney M). Data presented as mean ± SD: **P* < 0.05 by unpaired, two-tailed Student's T-test (*n* = 5/group) | | | | | | | | | |
|  |  |  |  |  |  |  |  |  |  |

**Supplemental Figure S1. Liver glycogen and triglyceride content and plasma FFA, β-hydroxybutyrate, TG and total cholesterol following 4 weeks dapagliflozin treatment in obese Zucker rats**

Male obese Zucker rats (fa/fa) were treated with either vehicle (● circles) or dapagliflozin (1mg/kg/day, ■ squares) for 4 weeks. A blood sample was collected in conscious animals following a 16 h fast. Following blood collection, the animals were anaesthetized and liver tissue was collected. Liver **(a)** glycogen and **(b)** triglyceride (TG) content. Fasting plasma (c) FFA and (d) β-hydroxybutyrate, (e) TG and (d) total cholesterol levels. Data presented as individual data points (*n* = 5-7/group). ** *P*<0.01 and ****P*<0.001, unpaired students t-test.

**Supplemental References**

1. Oakes, N. D., A. Kjellstedt, P. Thalen, B. Ljung, and N. Turner. 2013. Roles of Fatty Acid oversupply and impaired oxidation in lipid accumulation in tissues of obese rats. *J Lipids* **2013**: 420754.

2. Oakes, N. D., A. Kjellstedt, G. B. Forsberg, T. Clementz, G. Camejo, S. M. Furler, E. W. Kraegen, M. Olwegard-Halvarsson, A. B. Jenkins, and B. Ljung. 1999. Development and initial evaluation of a novel method for assessing tissue-specific plasma free fatty acid utilization in vivo using (R)-2-bromopalmitate tracer. *Journal of Lipid Research* **40**: 1155-1169.
